# The Destructive Static Tree-Pulling Test Provides Reliable Estimates of the Soil–Root Plate of Eastern Baltic Silver Birch (*Betula pendula* Roth.)

**DOI:** 10.3390/plants11111509

**Published:** 2022-06-04

**Authors:** Oskars Krišāns, Roberts Matisons, Jānis Vuguls, Andris Seipulis, Valters Samariks, Renāte Saleniece, Āris Jansons

**Affiliations:** Latvian State Forest Research Institute ‘Silava’, 111 Rigas Str., LV-2169 Salaspils, Latvia; oskars.krisans@silava.lv (O.K.); roberts.matisons@silava.lv (R.M.); janis.vuguls@silava.lv (J.V.); andris.seipulis@silava.lv (A.S.); valters.samariks@silava.lv (V.S.); renate.saleniece@silava.lv (R.S.)

**Keywords:** soil–root plate, silver birch, *Betula pendula*, windthrow, static tree-pulling

## Abstract

Under the intensifying cyclonic activity, the wind resistance of European forests could be increased through science-based adaptive forest management, which requires the quantification of tree stability. In this regard, the dimensions of the soil–root plate can be directly attributed to tree wind resistance; however, naturally uprooted trees might be a biased source of information for the evaluation of adaptive measures due to uncontrolled conditions and uneven sample size. Therefore, the dimensions of the soil–root plates of naturally windthrown silver birch trees (*Betula pendula* Roth.) are compared to artificially overturned trees under a static tree-pulling test in Eastern Baltic region. The application of static tree-pulling overestimated the dimensions of the soil–root plates of silver birch compared to windthrown trees. The overestimation of soil–root plate dimensions was consistent spatially and across soil types, which is likely a regional adaptation to local wind climate. This implies that static tree-pulling is representative of the assessment of the effects of adaptive management on tree stability via the dimensions of the soil–root plates.

## 1. Introduction

In the Eastern Baltic region, forest stands are subjected to an increasing frequency of wind disturbances due to the intensifying cyclonic activity from late autumn to early spring [1,2,3,4]. Wind damage is further amplified by the shortening of frozen soil periods [5,6,7], which leads to substantial economic losses [8], thus emphasizing the necessity for science-based climate-smart forest management [9,10,11,12,13]. In this regard, the properties of the soil–root plate are informative proxies of tree wind stability, allowing for a quantification of the effects of adaptive forest management [14,15]. Although the properties of the soil–root plate can be assessed for naturally windthrown trees, such trees can rarely form a reasonable sample size to evaluate the effects of management, as wind damage is largely stochastic [15]. Another option is experimental overthrowing by static tree-pulling tests, which have been widely applied to assess the tree mechanical stability [16,17,18]. Under static pulling, the tree is subjected to a continuous loading until a fatal failure as stem breakage or uprooting occurs; however, such an evaluation might result in biased estimates of the properties of the soil–root plate due to the lack of dynamic components of stability, such as inertia and swaying [19]. Accordingly, the assessment of the relationships between properties of the soil–root plate acquired by static tree-pulling tests and after windthrow are essential for a reliable evaluation of adaptive management [15]. Tree populations adapt to regional (e.g., wind climate) and local (e.g., soil) growing conditions [14,15]; hence, local data are necessary to reduce the bias when estimating tree wind loading resistance.

The aim of the present study is to compare the soil–root plates of middle-aged trees uprooted by windthrow and static tree-pulling tests on freely draining mineral, drained deep peat, and waterlogged mineral soils. Silver birch (*Betula pendula* Roth.), which is widespread on mineral and organic soils [20] and has high ecological and economic importance in North-East Europe [21], was used as a model species. Birch is reported to have a high plasticity of wind loading adaptation across a wide gradient of soil types [22]. Accordingly, we hypothesize that the static tree-pulling test overestimates the soil–root plates compared to windthrown trees.

## 2. Results and Discussion

**Figure 1:**
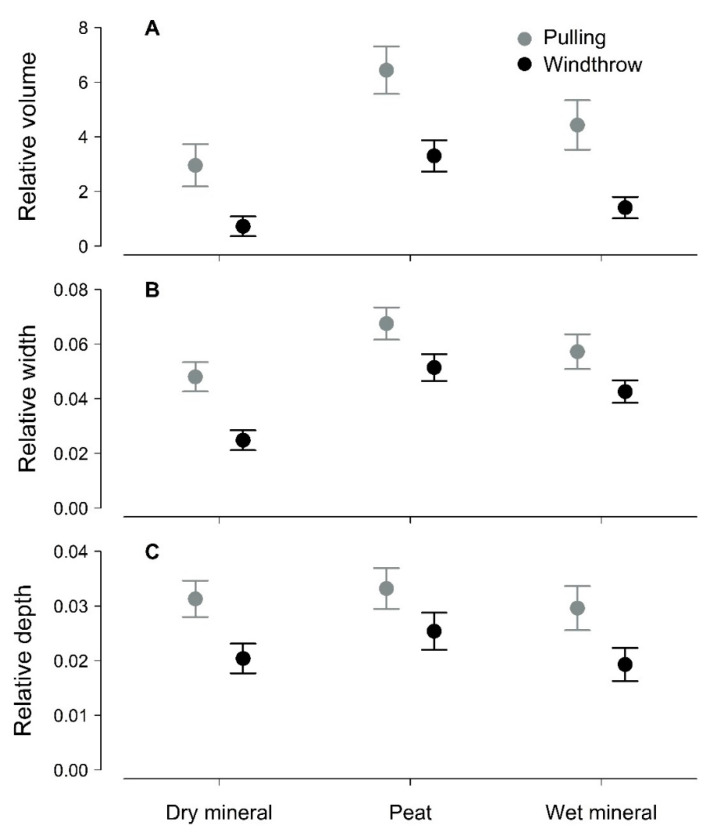
Estimated marginal mean (±95% standard error) volume (**A**) (expressed per stemwood volume), depth (**B**), and width (**C**) (expressed per stem diameter at breast height) of soil–root plates of pulled and windthrown trees of Eastern Baltic silver birch on freely draining mineral, drained deep peat, and periodically waterlogged mineral soils.

In the Eastern Baltic region, the application of static-pulling overestimated the dimensions of soil–root plates of middle-aged silver birch compared to windthrown trees (Figure 1; Table 1). The differences were strong (Wald’s *χ^2^* ≥ 11.80) and highly significant (*p* < 0.001) for the volume, depth, and particularly for width of the soil–root plate. The overestimation was spatially consistent, as indicated by a non-significant (*p* > 0.22) effect (random) of the studied sub-regions (the geobotanical district [23]) on the differences of soil–root plate dimensions, as determined by mixed-effect models (Table 1). The overestimation of soil–root plate width and depth was, on average, 53% and 46%, respectively, while for volume, which is a function of the two, it was 206%. The overestimation differed by soil type; it was lower for drained deep peat compared to periodically waterlogged mineral and particularly on freely draining mineral soils (52%, 101%, and 152%, respectively).

**Figure 2:**
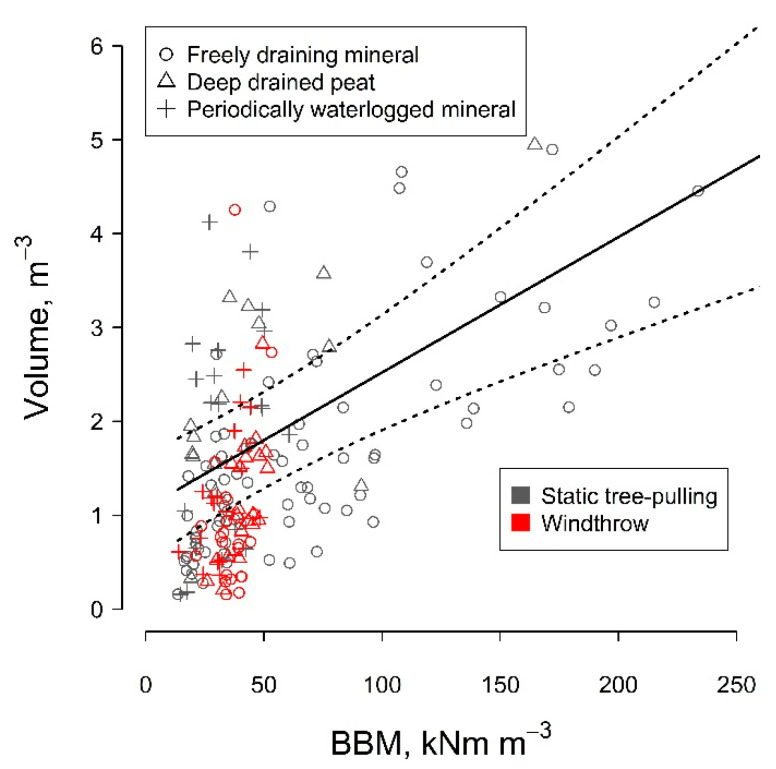
The relationship between relative stem basal bending moment (BBM in kNm) (expressed per stemwood volume in m^3^) and the volume of soil–root plate (in m^3^) of pulled and windthrown trees of Eastern Baltic silver birch on freely draining mineral, drained deep peat, and periodically waterlogged mineral soils.

Under static loading, larger soil–root plates were torn out, likely due to the lack of dynamic components of tree stability, such as tree swaying and inertia of soil [19], which was particularly prevalent for mineral soils (Figure 1). Accordingly, a larger amount of soil remained attached to the roots [24]. Additionally, due to a slower course of uprooting under static loading, root breakage occurred at smaller diameters [25], hence at a greater distance from the stem, thus explaining the explicit overestimation of soil–root plate width. Alternatively, the overestimation of the dimensions of the soil–root plate might be partially related to the specific selection of sample trees for the static tree-pulling, as vital, dominant trees without visual signs of mechanical damage were tested [18,22]. In contrast, the set of studied windthrown trees likely contained the weakest individuals in the stands, probably due to various reasons, such as lower vitality, which might include pathogen infestation [26,27]. For instance, root-rot is known to reduce the spread of the soil–root plate [28]; although, the decay of structural roots was not visible for the windthrown trees. The relationship between the basal bending moment at the fatal failure (BBM) and the volume of the soil–root plate for the pulled trees was linear, irrespective of soil type, though with some regional specifics (Figure 2, Table 2). For the windthrown trees, such a relationship was not assessed to avoid bias, as BBM could only be roughly estimated. However, the estimates of BBM of windthrown and pulled trees overlapped with the exception of freely draining mineral soils, where windthrown trees appeared smaller and weaker (Figure 2).

Silver birch is reported to have a high adaptability of soil–root plate to diverse soil conditions [16,29,30], for instance, plastically increasing leverage and rooting depth on loose soils [22,24]. Accordingly, soil type (freely draining mineral, drained deep peat, and waterlogged mineral) had a significant (*p* < 0.001) effect on the soil–root plate volume and surface radii (width) for both static tree-pulling and windthrow trees (Figure 1; Table 1 and Table 2). Although tested soil types have different mechanical properties [31,32,33], the absence of interaction between uprooting cause and soil type (*p* = 0.31) implied a consistent overestimation of soil–root plate by static tree-pulling across the studied soil types. Such a consistency can be related to the regional adaptation of Eastern Baltic silver birch to the local wind climate [34], supporting the adaptive significance of tree mechanical stability [35]. The consistency of deviations between uprooting cause, regardless of local/regional climate, landscape, and soil conditions, supports the comparability of the dimensions of the soil–root plates between static tree-pulling and windthrow. Thereby, the current results support static tree-pulling as a reliable approach for the assessment of the relationships between the properties of the soil–root plate, which is essential for a sufficient evaluation of adaptive management [15].

## 3. Materials and Methods

### 3.1. Data Acquisition and Measurements

Data for the soil–root plates overturned by static tree-pulling were acquired from earlier studies assessing the mechanical stability of Eastern Baltic silver birch [18,22] in the hemiboreal forests of the Eastern Baltic region in Latvia (55°56′–57°27′ N and 21°40′–26°23′ E; Table 3). The climate in the studied region is humid continental [36], influenced by the dominant westerlies from the North Atlantic [37], under which continentality increases eastwards [38]. The mean annual air temperature and the mean maximum wind speed are higher at the elevation of 10 m and in the western part of Latvia, reaching +7.9 °C and 27.5 m s^−1^, respectively, while in the eastern part, they are +6.3 °C and 11.9 m s^−1^, respectively [39,40]. In both coastal and inland areas, the warmest month is July (+17.8 and 17.7 °C, respectively) and the coldest is February (−2.0 and −4.4 °C, respectively) [39]. The mean annual sum of precipitation is 685.6 mm, with the highest monthly mean in August (94.7 mm) [39].

In total, data for 115 birch trees were collected in the destructive static tree-pulling tests (Table 3), which were conducted during 2019–2021 [18,22]. During the tests, the dimensions of the stem and crown were recorded and the BBM of the stem at the failure was estimated. In these studies, sample trees were located in 17 naturally regenerated silver birch stands with stand ages being 30–60 years, which predominantly contained spruce in the understory (advanced growth). The admixture of light-demanding species in the canopy was low, reaching 30% of the stand basal area. Three to ten trees were sampled per stand. The studied stands were growing on mesotrophic freely draining mineral (*Hylocomiosa* forest type with podzols), periodically waterlogged (wet) mesotrophic mineral soils (*Myrtilloso*-*sphagnosa* forest type with gleyic podzol) and eutrophic drained deep peat soils (*Myrtillosa* turf.mel. and *Oxalidosa* tuf. Mel. forest types with fibric histosols) [41,42]. The topography was flat.

For the representation of the dimensions of the soil–root plate of silver birch after natural uprooting, 62 freshly windthrown trees were surveyed across the territory of Latvia following cyclonic wind events under non-frozen soil conditions during January–March 2022 (Table 3). During the wind event, the maximum speed of wind (westerlies) ranged from 17.6–27.8 m s^−1^ with the strongest winds being recorded in the coastal western part of Latvia [39]. Only freshly windthrown trees were assessed to reduce the bias due to soil loosening and the erosion of soil–root plates. Similar to pulling tests, trees growing on the edges of openings were avoided. The surveyed trees represented 15 stands similar to those sampled during the static tree-pulling [18,22]. Canopy trees were surveyed, which represented the mean dimensions of stands and all of them formed the canopy. The surveyed stands on dry mineral and drained deep peat soils were mostly pure or admixed with Norway spruce (*Picea abies* (L.) H. Karst.). In the stands on wet mineral soils, birch was generally admixed by common aspen (*Populus tremula* L.). All of the studied stands were conventionally managed, which implies natural regeneration after a clear cut, with a rotation period of 71 years, and two to three thinnings. Such an approach intends to target a stand density of 2000–2500 trees per hectare after pre-commercial thinning, with a further consecutive reduction in the thinnings to 1000–1500 and 600–800 trees per hectare. Accordingly, all of the stands underwent pre-commercial thinning; however, no recent thinnings (5–10 years) have been conducted.

For all trees, the dimensions of the soil–root plate were measured by the same methodology. In brief, for each soil–root plate, five radii (at 0°, 45°, 90°, 135°, and 180°) of the surface were measured (Supplementary Materials, Figure S1). The maximum depth of soil–root plates was estimated by piercing a steel rod near the stem base perpendicular to the surface.

### 3.2. Data Analysis

The volume of the soil–root plate, which is the proxy of tree stability [25], was estimated as the volume of an elliptical paraboloid as follows:

(1) $$V=\left(\frac{1}{2}\right)\cdot \pi \cdot a\cdot b\cdot h,$$

where *h* is the depth, and *a* and *b* are the longest and shortest of the five measured radii of the soil–root plate, respectively (Supplementary Materials, Figure S1). Due to the differences in sizes of the studied trees (Table 3), relative dimensions of the soil–root plate were calculated. The relative volume of the soil–root plate was expressed per stemwood volume, yet relative width and depth of soil–root plate per diameter at breast height. Stemwood volume was calculated using local equation [43]:

*V* = 0.0000909·*H*^0.71677^*·DBH*^0.072*·ln(H)+*1.7570^ (2)

where *H* is the tree height and *DBH* is the stem diameter at breast height.

For the windthrown trees, *BBM* was approximated according to Jones (1984) [44] as:

*BBM* = 0.25· *π*·*C* ·*u*^2^·*p*/(*R*·*T*)·*w*·*h*, (3)

where *C* is a dimensionless species-specific drag coefficient (0.12); *u*, *p*, and *T* are the maximum wind speed, atmospheric pressure, and temperature during the strongest wind event in January–March 2022 recorded by the nearest meteorological station (less than 40 km); and *w* and *h* are the width and height of the canopy, respectively.

The differences in the soil–root plate according to the uprooting cause and soil type were assessed using a linear mixed-effects model:

*y_ij_* = *µ* + *uc_ij_* + *s_ij_* + *uc_ij_* × *s_ij_* + (*d_j_*) + (*uc_ij_* | *d_j_*) + *ε*, (4)

where *y_ij_* is the relative dimension of the soil–root plate (volume, depth, and width), *uc_ij_* is the fixed effect of the uprooting cause (windthrow or static tree-pulling test), *s_ij_* is the fixed effect of soil type, *uc_ij_* *× s_ij_* is the interaction between uprooting cause and soil type, and (*d_j_*) and (*uc_ij_* | *d_j_)* are the random intercept and random slope of uprooting cause by the local geobotanical district [23]. Geobotanical districts are local divisions of growing conditions according to edaphic and climatic factors, which affect the local adaptation of Eastern Baltic silver birch [35].

The relationship between BBM and the volume of soil–root plate was assessed using a linear mixed-effects model:

*v_ij_* = *µ* + *bbm_ij_* × *s_ij_* + *s_ij_* + (*d_j_*) + *ε*, (5)

where *v_ij_* is the volume of soil–root plate, *s_ij_* is a fixed effect of soil type, *bbm_ij_* *× s_ij_* is the fixed interaction of stem basal bending moment and soil type, and (*d_j_*) is the random intercept of the local geobotanical district. The significance of fixed effects was estimated by Wald’s *χ^2^* test; the leave-one-out test [45] was used to determine the significance of random effects. Data analysis was performed in R software (v 4.1.0.) (R Core Team, Vienna, Austria) [46] using the packages “MuMIn” [47], “lmerTest” [45], “emmeans” [48], and “lme4” [49].

## 4. Conclusions

Static pulling consistently overestimated the dimensions of the soil–root plate of Eastern Baltic silver birch, regardless of landscape and soil type, approving the hypothesis of the study. Such a systematical overestimation implies the comparability of soil–root plate dimensions acquired by static tree-pulling across environmental gradients and management practices. Despite the local data, such relationships likely apply to a wider geographic range, at least as a methodological development. Therefore, static tree-pulling can be encouraged to be used in the assessment of tree stability as a highly informative method.

## Figures and Tables

**Table 1:** Strength (Wald’s *χ*^2^) and significance (*p*-value) of the fixed effects of uprooting cause and soil type, as well as random variances and significance of the geobotanical district and model performance (R^2^) for the volume (expressed per stemwood volume), depth, and width (expressed per stem diameter at breast height) of soil–root plate of Eastern Baltic silver birch on freely draining mineral, drained deep peat, and periodically waterlogged mineral soils.

	Relative Volume		Relative Depth		Relative Width	
**Fixed Effects**	***χ^2^***	***p*-Value**	***χ* ^2^**	***p*-Value**	***χ* ^2^**	***p*-Value**
(Intercept)	25.97	<0.001	238.97	<0.001	202.73	<0.001
Uprooting cause	11.21	<0.001	11.80	<0.001	28.86	<0.001
Soil type	52.68	<0.001	2.37	0.30	42.60	<0.001
Uprooting cause by soil type	2.34	0.31	0.89	0.64	3.50	0.17
**Random Effects**	**Var.**	***p*-Value**	**Var.**	***p*-Value**	**Var.**	***p*-Value**
Geobotanical district:						
(Intercept)	0.86	0.23	1.12 × 10^−5^	0.48	3.13 × 10^−5^	0.95
Uprooting cause (wind)	0.87	0.22	1.65 × 10^−5^	0.42	2.17 × 10^−6^	0.96
Residual	2.38		4.03 × 10^−5^		9.48 × 10^−5^	
N_trees_	177		176		178	
N_district_	8		8		8	
Marginal R^2^	0.42		0.31		0.48	
Conditional R^2^	0.53		0.46		0.60	

**Table 2:** Strength (Wald’s *χ*^2^) and significance (*p*-value) of the fixed effects of stem basal bending moment (BBM) and soil type, random variances and significance of the geobotanical district and model performance (R^2^) for the volume of soil–root plate of Eastern Baltic silver birch on freely draining mineral, drained deep peat, and periodically waterlogged mineral soils.

	Volume	
**Fixed Effects**	***χ* ^2^**	***p*-Value**
BBM	65.95	<0.001
Soil type	20.21	<0.001
BBM by soil type	1.72	0.42
**Random Effects**	**Var.**	***p*-Value**
Geobotanical district:		
(Intercept)	0.17	0.02
Residual	0.71	
N_trees_	115	
N_district_	3	
Marginal R^2^	0.40	
Conditional R^2^	0.52	

**Table 3:** Tree (Tree n) and stand number (Stand n); mean (±standard error) stem diameter at breast height (DBH); height (H), ratio of height, and stem diameter at breast height (H/DBH); stemwood volume (V_stem_); depth, width, and volume (V_roots_) of soil–root plate of pulled and windthrown Eastern Baltic silver birches on freely draining mineral, drained deep peat, and periodically waterlogged mineral soils.

Soil	Tree n	Stand n	DBH (cm)	H (m)	H/DBH	V_stem_ (m^3^)	Width (m)	Depth (m)	V_roots_ (m^3^)
**Pulled**									
Dry	79	10	25.9 ± 0.8	27.3 ± 0.5	1.09 ± 0.02	0.75 ± 0.06	1.1 ± 0.1	0.7 ± 0.1	1.66 ± 0.13
Peat	15	3	21.0 ± 1.2	22.5 ± 0.6	1.10 ± 0.04	0.38 ± 0.06	1.4 ± 0.1	0.7 ± 0.1	2.31 ± 0.30
Wet	21	4	22.0 ± 0.9	22.0 ± 0.7	1.01 ± 0.02	0.41 ± 0.04	1.3 ± 0.1	0.7 ± 0.1	1.97 ± 0.25
**Windthrown**									
Dry	22	10	34.5 ± 1.7	29.0 ± 0.8	0.86 ± 0.03	1.32 ± 0.16	0.9 ± 0.1	0.7 ± 0.1	0.93 ± 0.20
Peat	22	1	21.4 ± 0.9	23.0 ± 0.5	1.10 ± 0.03	0.40 ± 0.04	1.1 ± 0.1	0.6 ± 0.1	1.42 ± 0.28
Wet	18	4	29.4 ± 1.6	28.8 ± 0.4	1.03 ± 0.06	0.93 ± 0.10	1.2 ± 0.1	0.5 ± 0.1	1.15 ± 0.16

## Data Availability

Not applicable.

## Supplementary Materials

The following supporting information can be downloaded at: https://www.mdpi.com/article/10.3390/plants11111509/s1; Figure S1: Five radii on the surface of soil–root plate (0°, 45°, 90°, 135°, and 180°).
