# Supplementary figures and images for: The Destructive Static Tree-Pulling Test Provides Reliable Estimates of the Soil–Root Plate of Eastern Baltic Silver Birch (Betula pendula Roth.)
Source: Plants (Basel). 2022 Jun 4;11(11):1509. doi: 10.3390/plants11111509 (PMC9182673; doi:10.3390/plants11111509)

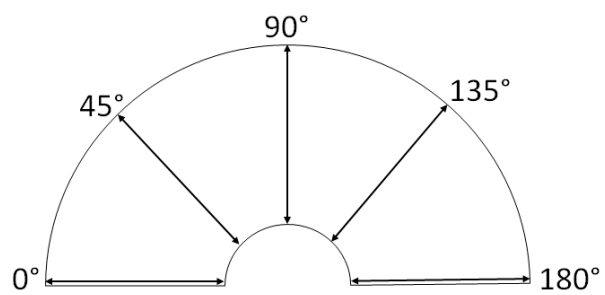

**Figure S1.** Five radii on the surface of soil-root plate (0°, 45°, 90°, 135°, and 180°).

Supplement: Supplementary file 1 [file plants-11-01509-s001.zip › plants-1736170-supplementary.pdf]
